# Intramolecular and Intermolecular Interaction Switching in the Aggregates of Perylene Diimide Trimer: Effect of Hydrophobicity

**DOI:** 10.3390/molecules28073003

**Published:** 2023-03-28

**Authors:** Peiyuan Su, Guangliu Ran, Hang Wang, Jianing Yue, Qingyu Kong, Zhishan Bo, Wenkai Zhang

**Affiliations:** 1 Department of Physics and Applied Optics Beijing Area Major Laboratory, Center for Advanced Quantum Studies, Beijing Normal University, Beijing 100875, China; 2 Synchrotron Soleil, L’Orme des Merisiers, 91190 Saint-Aubin, France; 3 College of Textiles & Clothing, Qingdao University, Qingdao 266071, China

**Keywords:** PDI trimer, aggregates, hydrophobic effect, transient absorption spectroscopy

## Abstract

The research on perylene diimide (PDI) aggregates effectively promotes their applications in organic photovoltaic solar cells and fluorescent sensors. In this paper, a PDI fabricated with three peripheral PDI units (N, N’-bis(6-undecyl) perylene-3,4,9,10-bis(dicarboximide)) is investigated. The trimer shows different absorption and fluorescence properties due to hydrophobicity when dissolved in the mixed solvent of tetrahydrofuran (THF) and water. Through comprehensive analysis of the fluorescence lifetime and transient absorption spectroscopic results, we concluded that the trimer underwent different excited state kinetic pathways with different concentrations of water in THF. When dissolved in pure THF solvent, both the intramolecular charge-transfer and excimer states are formed. When the water concentration increases from 0 to 50% (*v*/*v*), the formation time of the excimer state and its structural relaxation time are prolonged, illustrating the arising of the intermolecular excimer state. It is interesting to determine that the probability of the intramolecular charge-transfer pathway will first decrease and then increase as the speed of intermolecular excimer formation slows down. The two inflection points appear when the water concentration is above 10% and 40%. The results not only highlight the importance of hydrophobicity on the aggregate properties of PDI multimers but also guide the further design of PDI-based organic photovoltaic solar cells.

## 1. Introduction

Perylene-3,4,9,10-tetracarboxylic acid diimides (perylene diimides, PDIs) have been attractive candidates for the application of organic field effect transistor (OFET) [1,2], light-emitting diodes (LED) [3,4] and organic solar cells (OSC) [5,6,7,8] because of their high electrical conductivity and fluorescence quantum efficiency, strong visible light absorption coefficient as well as excellent chemical stability [9,10,11]. According to the structure of PDI, changing the substituents at different positions, such as the imide or the bay positions, can modify the properties of PDI and obtain various derivatives for a wide range of applications [12]. However, the performances of these optical devices depend strongly on interchromophoric interactions [13,14]. Therefore, it is of great importance to understand how these interactions impact the properties, such as absorption, fluorescence, exciton and electron transport, of bulk solid-state materials [15]. Such understanding can be derived from investigations on supramolecular aggregates that constitute the intermediate state between monomer and solid-state materials [16,17]. The most common PDI aggregates are H-type, which is the face-to-face aggregation of PDI chromophores and usually leads to blue-shift of the molecular absorption [18], and J-type, which arises from the head-to-tail aggregation of chromophores and usually leads to red-shift of the molecular absorption [19]. The PDI monomers could be linked covalently or with spacers to form different aggregation states [20,21,22]. By modifying the polarity of the solvent, the non-covalent π–π stacking between PDI chromophores can also be realized to induce different aggregates [23,24,25].

In addition, the water-induced hydrophobic effect is also an effective way to form PDI aggregates with different properties [26,27,28]. In 2012, Chen et al. investigated 17 solvents, including water, and discovered a unique hydrophobic effect on PDI aggregations [29]. PDI molecules with various side chains of appropriate lengths are shown to form aggregates easily with a higher quantum yield under the influence of hydrophobicity [30,31,32].

Desirable optical or electronic properties that are not given by the individual unit, such as the generation of symmetry-breaking charge separate (SB-CS) state [33,34,35], singlet fission (SF) state [36,37,38] and excimer state [39,40,41,42,43] along the π-stacks, are observed in aggregates. SB-CS, SF and excimer states are favorable to improve the efficiency of OSCs [44,45]. Recently, our group discovered that the formation of an SB-CS state in PDI-C5 due to the hydrophobic effect can be induced by adding a proper proportion of water [46]. Following from this, we use the acetylene and benzene ring to connect two PDI-C5 to form a dimer and find the excimer formation in higher polar solvents (such as acetone) due to the aggregation [39]. As the molecular structure has a great influence on intramolecular and intermolecular dynamics, we further turned our study on PDI-C5 trimers. Understanding how solution and molecular structure affects the aggregate properties with different time-resolved spectroscopy methods, such as time-resolved fluorescence or absorption spectroscopy, could help us analyze the charge-transfer process in PDI and its derivatives. Previously, a PDI trimmer with slipped ‘‘face-to-face’’ stacked structure has been reported to form the excimer state [47]. Recently, a time resolved transient absorption spectroscopy study based on a PDI trimer with a twisted structure proves the formation of SF states [38]. Two other studies based on a covalent slip-stacked structure and a rigid triangular architecture give insights into the electronic states mixing of excimer and SB-CS states [48,49].

**Figure 1:**
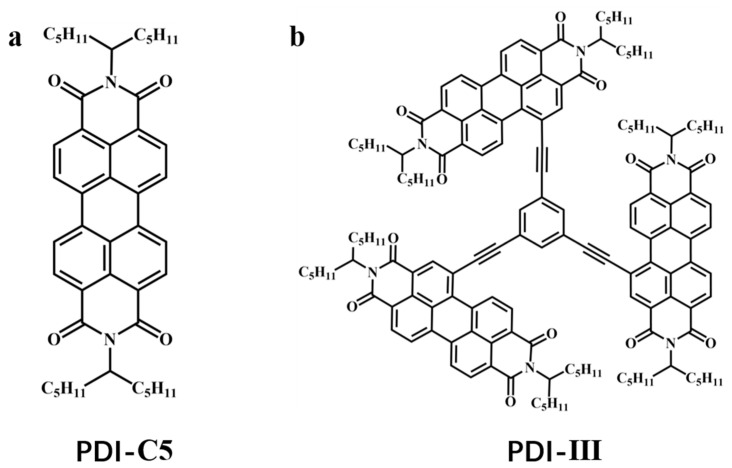
Structures of PDI-C5 (**a**) and PDI-III (**b**).

This paper mainly investigates the hydrophobic effect on a PDI trimer (PDI-III) formed by connecting three PDI-C5 molecules to the central benzene with the acetylene spacer. The structure of the trimer and its monomer unit PDI-C5 are shown in Figure 1. The hydrophobicity effect on PDI-III was investigated in THF solution with different water volume ratios, from 10–50%. By analyzing the Ultraviolet–Visible (UV-Vis) absorption and fluorescence spectra of the sample solutions with different water concentrations, we classified the aggregates of PDI-III into H-type. The combined transient absorption spectra and global and target analysis illustrate that the molecule is likely to form excimer and charge-transfer (CT) states under the influence of hydrophobicity. As the water concentration increases, the CT state remains unchanged, while the excimer shifts from an intramolecular state to an intermolecular state. The relationship between the excimer and CT states shows that the occupied percentage of the CT state will first decrease and then increase, depending on the formation rate and the structural relaxation speed of the intermolecular excimer state. Finally, two inflection points are obtained when 10% and 40% water are added as the interactions between intramolecular chromophores and intermolecular chromophores are switching. This PDI trimer exhibits a higher photovoltaic conversion efficiency when assembled as an electron acceptor in an organic solar cell with PBDB-T compared to its corresponding dimer and multimer [50]. Our study of the PDI-III aggregates may guide a better understanding of the CT process of PDI derivatives in OSCs.

## 2. Results and Discussions

**Figure 2:**
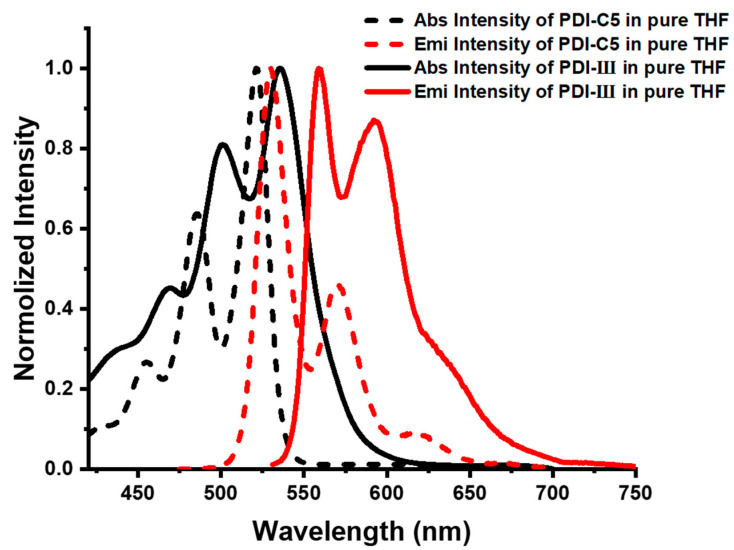
Normalized absorption and fluorescence spectra of PDI-III and PDI-C5 in pure THF.

The UV-Vis absorption and fluorescence spectra of PDI-III and PDI-C5 in pure THF are shown in Figure 2. It can be found that the absorption spectra of PDI-III (black solid line) still have good vibrationally resolved characteristics despite the formation of the trimeric structure through the PDI-C5 monomers. The PDI trimer’s absorption peaks of 0-1, 0-2 and 0-3 correspond to 536 nm, 500 nm and 468 nm, respectively. Comparing those with PDI-C5 (black dash line), the absorption peaks of PDI-III have a particular redshift, and the ratio of 0-1/0-2 peak intensity drops from 0.8 to 0.6 as calculated from Figure 2. The fluorescence quantum efficiencies of PDI-C5 and PDI-III in THF are 0.72 and 0.18, respectively. Alkyne chain linkages that lead to the growth of conjugated structures might be the reason why the absorption peaks of PDI-III are redshifted when compared with that of PDI-C5 [34]. Moreover, the attenuated 0-1/0-2 absorption peak intensity and the much lower fluorescence quantum efficiency prove that H-type aggregation, rather than J-type aggregation, occurs in the pure THF solvent of PDI-III [47,51,52]. It can also be seen from Figure 2 that the absorption and fluorescence spectra of PDI-III (black and red solid lines) are in good mirror symmetry at the peaks of vibrational energy transitions 0-1 and 0-2. A faint fluorescence peak appears at the emission wavelength greater than 650 nm. The reduction of quantum efficiency indicates that there may be other excited state relaxation paths for the trimer in addition to the conventional charge-transfer relaxation process.

**Figure 3:**
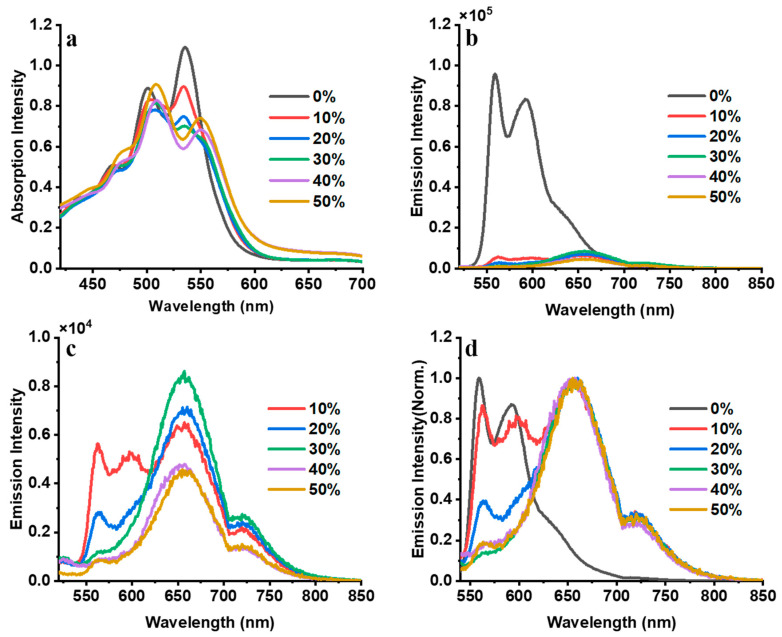
The absorption (**a**) and fluorescence (**b**) spectra of PDI-III with different water concentrations; the fluorescence (**c**) and normalized fluorescence spectra (**d**) of PDI-III with different water concentrations (0% water is excluded).

To study the effect of hydrophobicity on the H aggregates of PDI-III, we prepared five different THF solutions by adding 10%, 20%, 30%, 40% and 50% water. THF is used in our experiment because it has very good inter-solubility with water. At the same time, the proper polarity of THF allows us to clearly and precisely distinguish how the addition of different proportions of water will affect the aggregation state of PDI-III [53,54]. The UV-Vis absorption and fluorescence spectra of PDI-III in these five solutions and the reference (without water) are shown in Figure 3. The changes of UV-Vis absorption spectra in Figure 3a show that the two absorption peaks of PDI-III corresponding to 0-1 and 0-2 transitions move from 536 nm and 500 nm to 551 nm and 508 nm, respectively, as the proportion of water keeps increasing. Simultaneously, the peak ratio of 0-1/0-2 becomes continuously smaller, which implies the formation of H-aggregates. As shown in Figure 3b, the fluorescence intensity of the aggregated state generated by this molecule is significantly reduced after adding water compared to the fluorescence spectrum of PDI-III without water, which further confirms the generation of H-aggregates.

Figure 3c shows the variation of the fluorescence spectra with different volume concentrations of water from 10% to 50%. The fluorescence emission wavelength of the PDI-III has a significant red shift and forms a broad fluorescence peak above 650 nm when the water concentration increases, which indicates the formation of excimer [20,21,36]. To acquire the locations of the fluorescence peaks, all fluorescence spectra are normalized as shown in Figure 3d. The original fluorescence emission peaks located at 564 nm and 600 nm due to the CT state relaxation can still be observed when the percentage of water is 10%, while the intensity drops drastically [33,34]. As the proportion of water keeps increasing, the fluorescence emission peak related to the CT state decreases quickly, and the new fluorescence peaks (650 and 725 nm) corresponding to the excimer reach the maximum almost simultaneously independent of the water concentration. The fluorescence peak intensity related to the excimer also decreased a little after the water proportion reached 40%. From the above spectral information, we suggest two different relaxation paths, the formation of the CT state and excimer state, respectively, in the aggregates of PDI-III under the influence of hydrophobicity. As the percentage of water increases, intermolecular interactions will significantly impact this excimer state, leading to intensity variation of the fluorescence signal. To verify our conjecture, we measured the fluorescence lifetime of the aggregates with different water concentrations.

**Figure 4:**
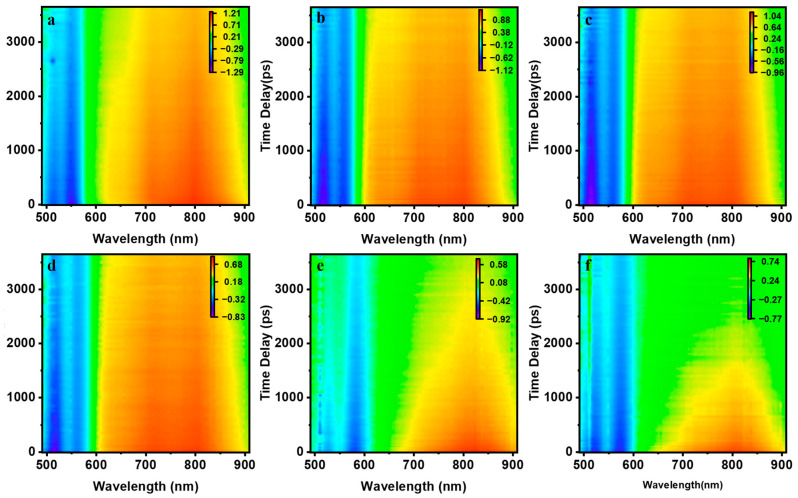
Two-dimensional (2D) transient absorption spectra of PDI-III with different water percentages: (**a**): 0%; (**b**): 10%; (**c**): 20%; (**d**): 30%; (**e**): 40%; and (**f**): 50%.

**Figure 5:**
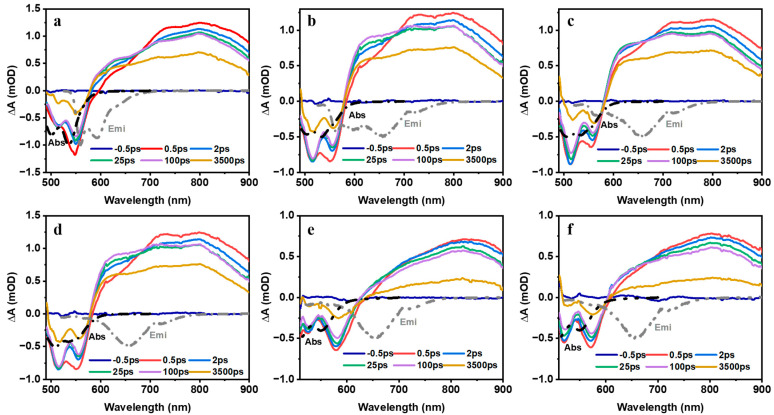
The transient absorption spectra of the PDI-III at different time delays with different water percentages: (**a**): 0%; (**b**): 10%; (**c**): 20%; (**d**): 30%; (**e**): 40%; and (**f**): 50%.

The fitting curves of fluorescence lifetime data with different water percentages added are shown in Figure S1, and the results are summarized in Table 1. It can be found that the fluorescence lifetime stays around 4 nanoseconds (ns) independent of the water concentrations. In contrast, the fluorescence quantum yield decreases under the influence of hydrophobicity. According to our analysis of the fluorescence signal, there should be two radiative relaxation paths for the aggregated states of PDI-III. However, we can only acquire one lifetime. There are two possible reasons why we can only obtain one lifetime value: (1) the generation of non-radiative relaxation processes in the excimer state makes the fluorescence intensity too weak to be measured [22,30]; (2) the extremely high fluorescence intensity of the CT state and the growth of its ratio overwhelm the signal of the excimer state. Transient absorption spectroscopies shown in Figure 4 and Figure 5 give a better depiction of the details of the two relaxation paths.

Figure 4a–f represent the PDI trimer’s two-dimensional transient absorption spectra without and with water percentages of 10%, 20%, 30%, 40% and 50%. All the transient absorption signals are divided into positive and negative parts bounded by 600 nm. Negative signals below 600 nm represent the ground-state bleaching (GSB) and stimulated emission (SE), while the positive signals above 600 nm represent the excited state absorption (ESA). All the negative signals have peaks around 520 nm and 570 nm, which are consistent with the positions of the corresponding absorption spectra in Figure 2. Meanwhile, positive signals of the six solutions are divided into three categories according to the number of positive peaks they have. Figure 4a shows two positive peaks at 700 and 800 nm, Figure 4b–d has an additional peak at 620 nm, while Figure 4e,f has only one positive peak at 800 nm.

To clearly show the variations of the transient absorption spectra under the influence of hydrophobicity, we depicted the transient absorption spectra of PDI-III solutions at select time delays when different volume ratios of water are added (Figure 5). The detailed transient absorption spectra of PDI-III solutions at all time delays are shown in Figure S2. Figure 5a shows the TA spectra of PDI-III at different time delays without water. Figure 5b–d represent the TA spectra of PDI-III when 10%, 20% and 30% of water are added, respectively. Figure 5e,f show the results of the TA spectra of PDI-III with 40% and 50% water concentrations, respectively. Although all the negative signals are located at 520 nm and 570 nm, their peak intensity varied with increasing water percentage. The decreasing trend of the intensity ratio (570 nm/520 nm) is consistent with the intensity ratio of the corresponding absorption peaks 0-0/0-1 except for the spectra of Figure 5e,f when 40% and 50% of water are added, respectively. The intensity ratios of the two negative signals (570 nm/520 nm) in the two solutions (40% and 50% water added) increase to more than 1, while the intensity ratios of the corresponding absorption peaks in Figure 3a decrease to 0.77 for the PDI-III solutions (40% and 50% water added). As depicted in Figure 5, the GSB signal corresponds to the absorption spectra in the black dashed curve and the SE signal corresponds to the fluorescence spectra in the gray dashed curve, which overlap at around 570 nm. The little difference in the intensity ratios of the two negative signals (570 nm/520 nm) lies in the fact that the fluorescence peak at 570 nm starts to become distinct when the water percentage reaches 40% (Figure 3c), which enhances the transient absorption signal of SE at 570 nm and thus breaks the decreasing trend of the intensity ratios of the two negative signals (570 nm/520 nm).

The ESA band adjacent to the GSB overlaps with the SE, resulting in three basin-like spectral features (BSF) within the ranges of 620–640 nm, 650–700 nm and 700–800 nm [20,42,52]. The three BSFs coincide with the fluorescence spectra (gray dashed curve) as shown in Figure 3. Comparing with the fluorescence spectra, the BSF located in the range of 620–640 nm corresponds to the fluorescence signal of the CT state. In contrast, the other two BSFs lie in 650–700 nm, and 700–800 nm corresponds to the fluorescence signal of the excimer state. In the early time delays before 25 ps (see Figure 5a), the BSF between 620 and 640 nm gradually decreases due to the radiative relaxation of the CT state, which results in the weakening of the intensity of its corresponding excited emission signal. The other two BSFs (650–700 nm and 700–800 nm) become relatively more apparent because of the longer radiative relaxation time of the excimer state [24,35]. The three BSFs decay simultaneously in the time range after 25 ps as shown in Figure 5a. In contrast, only the two BSFs due to the SE signal of the excimer state can be observed in Figure 5b–d when the percentage of water reaches 10%. When the ratio of water exceeds 40%, as shown in Figure 5e,f, the BSF caused by the SE of the CT state arises again. At the same time, the two BSFs generated by the SE signal of the excimer state become very weak. The results are not only in high agreement with our above analysis but also imply that there may be dynamic switching between the excimer and CT states due to hydrophobicity. Since the signals are superpositions of multiple transient absorption spectra, global and target analysis is used to unravel the details of the relaxation pathways of PDI-III.

**Figure 6:**
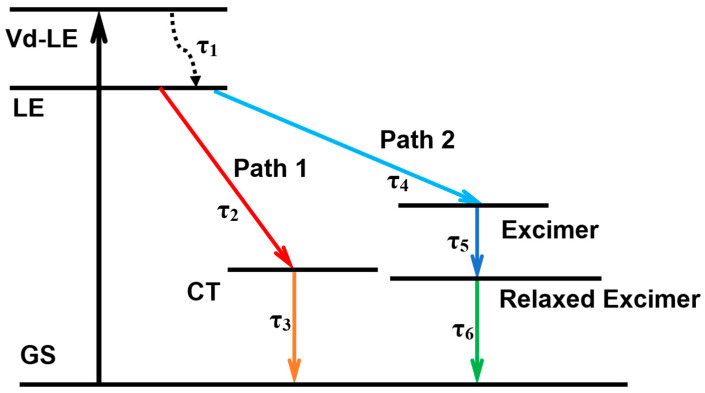
Constructed model for global and target analysis.

The established model is shown in Figure 6. Since the PDI-III has a vibrationally resolved energy level, 480 nm, which corresponds to the energy needed for the 0-2 transition, will excite PDI-III to the highest vibrational energy level, the vibrational dynamical-local excited (Vd-LE) state, which subsequently returns to the LE state through vibrational relaxation (τ_1_). According to our above analysis, PDI-III has two relaxation processes under hydrophobicity: transforming to the CT state(τ_2_) or the excimer state (τ_4_). The excimer tends to be stabilized by structural relaxation (τ_5_) [20,39], which is named a relaxed excimer state. Finally, the CT and relaxed excimer states will return to the ground state (GS) by radiative relaxation (τ_3_ and τ_6_, respectively). Through global and target analysis, we summarize the obtained lifetimes of all the processes in Table 2.

The vibrational relaxation lifetime of the Vd-LE state (τ_1_) stays around 500 fs, which is consistent with previous results [20,37,38,47]. As the percentage of water gradually increases, one lifetime of LE (τ_2_), which corresponds to the generation of the CT state, remains the same around a few picoseconds [24,34,42]. However, the other lifetime of LE (τ_4_), which corresponds to the formation of the excimer, begins to increase from a few picoseconds without water to tens of picoseconds and finally reaches more than 100 picoseconds when water is added. The lifetimes of the corresponding structural relaxation also increase from a few hundred picoseconds to a maximum of 1.2 ns. The radiative relaxation time of the final CT state (τ_3_) is about 4 ns, which is consistent with the fluorescence lifetime we obtained. In contrast, the radiative relaxation time of the excimer state stays in the tens of nanoseconds, which is also in high agreement with previous results [20,36,41,42]. By analyzing the lifetimes, we found that both the formation and the radiative relaxation time of the CT state remain essentially constant under the influence of hydrophobicity, which indicates that the formation of the CT state of this PDI trimer is always inside the structure, namely the intramolecular charge-transfer (ICT) state. In the case of the excimer state, both the formation and the structural relaxation time are prolonged with the increase of the percentage of water, which implies that the hydrophobicity strengthens the intermolecular interaction and influences the nature of the aggregation state of the PDI trimer. Ultimately, the excimer state between intramolecular chromophores is transferred to intermolecular chromophores with longer lifetimes. It has been demonstrated in many other studies that the enhancement of intermolecular interactions due to the external environment leads to the creation of intermolecular excimer states with longer formation time and structure relaxation time [55,56,57].

**Figure 7:**
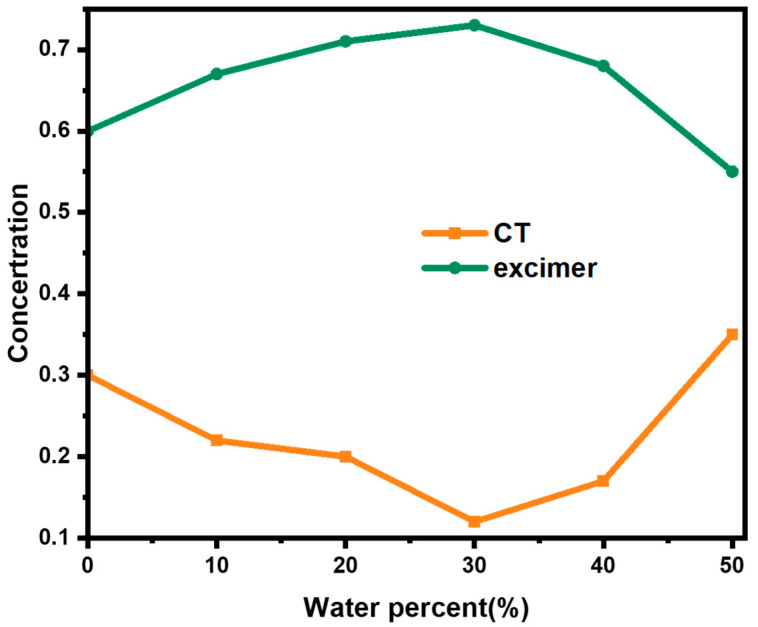
Concentration variations of the CT and excimer states at different water percentages.

We also obtained the species-associated decay spectra of each state (Figure S3) and the concentration variations of corresponding processes (Figure S4) from global and target analysis. Although most of the molecules are excited to the Vd-LE state, the concentrations of the LE state we get from Figure S4 are a little bit higher than that of the Vd-LE state. The difference lies in the fact that not all the PDI-III molecules have enough energy to be excited to the Vd-LE state. A small amount of them will be excited to the LE state directly. Therefore, the concentration of the LE state will be a little bit higher than that of Vd-LE state. The results in turn prove the accuracy of our model for global and target analysis since it is close to reality. Finally, the variations of concentration of the two states (CT state and excimer state) are summarized in Figure 7. It can be found that the ratio of the components (CT: excimer) is 1:2 without water addition. The formation of the intramolecular excimer state leads to a significant decrease in the fluorescence quantum efficiency of the PDI trimer. When the content of water increases to 10%, the ratio of the excimer state further increases due to the hydrophobicity-caused enhancement of intermolecular interactions, which transforms the intramolecular excimer state to the intermolecular excimer state, resulting in a further reduction of the fluorescence quantum efficiency. The percentage of intermolecular excimer states reaches its maximum when the percentage of water is 30% and starts to decrease when more water is added, probably due to the long formation time for intermolecular excimer (more than 100 ps), and then the competing reaction mechanism is favorable for the formation of ICT states in PDI-III. This is also the reason why we observed a decrease in the fluorescence intensity of the excimer state in Figure 3c. The results of the global and target analyses can not only help us to explain the changes in the fluorescence spectra and the appearance of the two turning points in the transient absorption spectra but also allow us to decipher the dynamic balance between the CT state and the excimer state under the effect of hydrophobicity. This research will provide valuable guidance in understanding the role of hydrophobicity on the aggregation states of PDIs.

## 3. Materials and Methods

### 3.1. Materials

The details of molecular structures, design, and synthesis methods of PDI-III can be found in the literature [50]. The materials and organic solvents were bought from certified commercial resources and used as received.

### 3.2. Steady-State Spectroscopy and Transient Absorption Spectroscopy

UV-Vis absorption spectroscopy was collected using commercial Agilent Cary 60 (Santa Clara, CA, USA) Emission spectroscopy and fluorescence lifetime were measured by commercial fluorescence spectrometer FLS980 (Livingston, UK). Transient absorption spectroscopy was obtained using a femtosecond transient absorption (Fs-TA) spectrometer (Harpia-TA, Light Conversion, Vilnius, Lithuania). Details of the instrument have been described elsewhere [39]. The samples are excited with 480 nm. The concentration was 10^−5^ mol/L for UV-Vis and Fs-TA and 10^−6^ mol/L for fluorescence. The fluorescence quantum yield was measured by the comparative method in the literature [33]. The global and target analysis was performed by the software Glotaran [58].

## 4. Conclusions

In this article, we investigated the effect of hydrophobicity on the PDI trimer’s aggregation state. By the analysis of UV-Vis absorption and fluorescence spectra, we first demonstrated that H-type aggregation is formed in the pure THF solution. Subsequently, the phenomenon of H aggregation became more and more distinctive due to the hydrophobic effect. By the lifetime analysis of the time-resolved fluorescence spectra and global and target fitting of transient absorption spectra, we suggest that H aggregation leads to the formation of two different states, the ICT state and excimer state. Moreover, we demonstrated that the charge-transfer state remains within the molecule since the rate of formation and the lifetime of its relaxation remain unchanged as the proportion of water increases. The formation time and relaxation lifetime of the charge-transfer state are a few picoseconds and four nanoseconds, respectively.

Meanwhile, the excimer state, on the other hand, shifts from intramolecular chromophores to intermolecular chromophores due to a slower rate of formation and a longer structural relaxation time. The results of the global and target analyses show that the component of the ICT state decreases first and then increases as the percentage of water in the solvent increases. The turning points appear when the percentage of water reaches 10% and 40%. To conclude, the PDI trimer’s hydrophobicity greatly influences the photoexcited relaxation pathways on its aggregates. Formation of the CT and the excimer states can be controlled by changing the water concentrations. Since CT and excimer formation are both efficient enhancements for charge separation in photovoltaic solar cells, this study can provide new perspectives for designing organic photovoltaic solar cell materials.

## Figures and Tables

**Table 1:** Fluorescence lifetimes of PDI-III in THF with different water percentages.

Water Percentage	Lifetime τ (ns)	Quantum Yield
0%	4.62 ± 0.1	0.18
10%	3.6 ± 0.1	0.02
20%	4.1 ± 0.1	0.02
30%	4.5 ± 0.08	0.02
40%	4.4 ± 0.03	0.01
50%	4.2 ± 0.02	0.01

**Table 2:** Lifetimes of PDI-III species in THF with different water percentages.

Water Percentage	Lifetime (τ)					
	τ_1_ (fs)	τ_2_ (ps)	τ_3_ (ns)	τ_4_ (ps)	τ_5_ (ns)	τ_6_ (ns)
0%	512 ± 27	7.2 ± 1	4.6 ± 0.7	5.8 ± 1	0.48 ± 0.1	12.3 ± 2.1
10%	526 ± 58	5.1 ± 1	4.4 ± 0.5	9.6± 1	0.7 ± 0.1	14.9 ± 1.5
20%	560 ± 80	7.5 ± 1	4.3 ± 0.7	29.8 ± 4	0.91 ± 0.1	18.7 ± 2.5
30%	540 ± 90	6.1 ± 1	4.5 ± 0.7	57.8 ± 9	1.17 ± 0.2	16.5 ± 2.7
40%	526 ± 58	9.1 ± 1	4.3 ± 0.5	102 ± 11	1.23 ± 0.2	17.9 ± 1.9
50%	568 ± 79	8.9 ± 1	4.1 ± 0.4	107.5 ± 12	1.22 ± 0.1	14.9 ± 1.6

## Data Availability

Data is available upon request.

## Supplementary Materials

The following supporting information can be downloaded at: https://www.mdpi.com/article/10.3390/molecules28073003/s1, Figure S1: The fluorescence lifetime fitting of the PDI trimer with 480 nm excitation; Figure S2: The transient absorption spectra of the PDI trimer at all time delays; Figure S3: SADS data of different species with different water percentages; Figure S4: The concentration variations of corresponding processes with different water percentages.

## Sample Availability

Samples are available upon request.
