# Supplementary material for: Intramolecular and Intermolecular Interaction Switching in the Aggregates of Perylene Diimide Trimer: Effect of Hydrophobicity
Source: Molecules. 2023 Mar 28;28(7):3003. doi: 10.3390/molecules28073003 (PMC10095916; doi:10.3390/molecules28073003)
Supplement: Supplementary file 1 [file molecules-28-03003-s001.zip › molecules-2260872-SI.pdf]

# Supporting information

# Intramolecular and Intermolecular Interaction Switching in the Aggregates of Perylene Diimide Trimer: Effect of Hydrophobicity

**Peiyuan Su [1,2,†], Guangliu Ran [1,†], Hang Wang [3], Jianing Yue [1], Qingyu Kong [2,*], Zhishan Bo [3] and Wenkai Zhang [1,*]**

[1] Department of Physics and Applied Optics Beijing Area Major Laboratory, Center for Advanced Quantum Studies, Beijing Normal University, Beijing 100875, China
[2] Synchrotron Soleil, L'Orme des Merisiers, 91190 Saint-Aubin, France
[3] College of Textiles & Clothing, Qingdao University, Qingdao 266071, China
* Correspondence: qingyu.kong@synchrotron-soleil.fr (Q.K.); wkzhang@bnu.edu.cn (W.Z.)
† These authors contributed equally to this work.

The fluorescence lifetime in Figure S1 is obtained by single exponential fitting in equation 1:

$$F(t) = A \cdot e^{-\frac{t}{\tau}} \qquad (1)$$

where A is the amplitude of the fluorescence intensity. $\tau$ is the lifetime that we can finally get.

Using the Glotaran program[1], we performed global and target analysis (GTA) by inserting our target model into the program. The model is expressed in equation 2:

$$\psi(\lambda, t) = \sum_{l=1}^{n_{comp}} C_l(t) \cdot S_l(\lambda) \qquad (2)$$

Where $C_l(t)$ and $S_l(\lambda)$ are unknown concentration profile and spectrum of components $l$, respectively. The species-associated decay spectra and concentration in Figures S2 and S3 are obtained from global and target analysis.

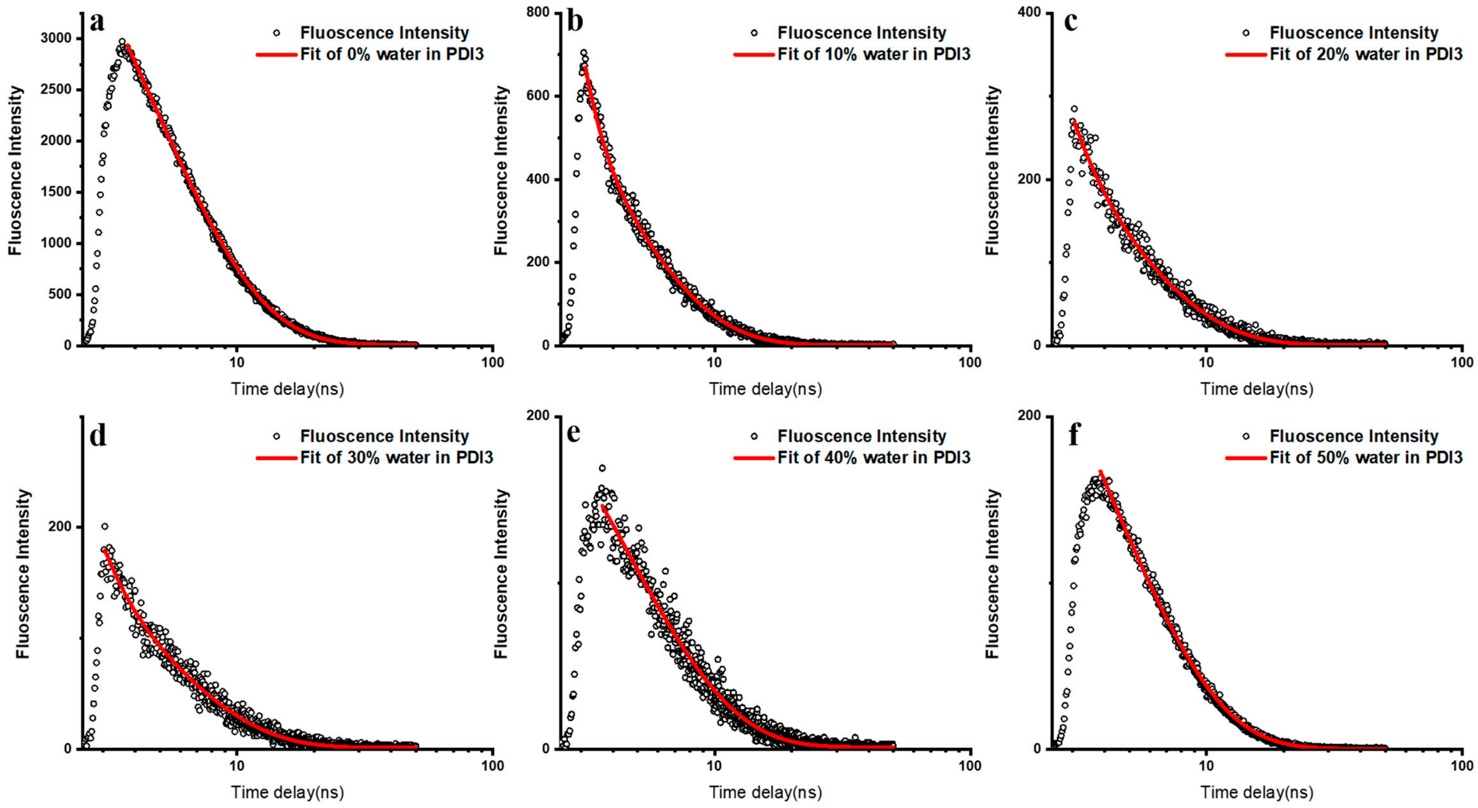


**Figure S1. The fluorescence lifetime fitting of PDI trimer solutions with 480nm exciation when different percentages of water added: a:0%; b:10%; c:20%; d:30%; e:40%; f:50%.**

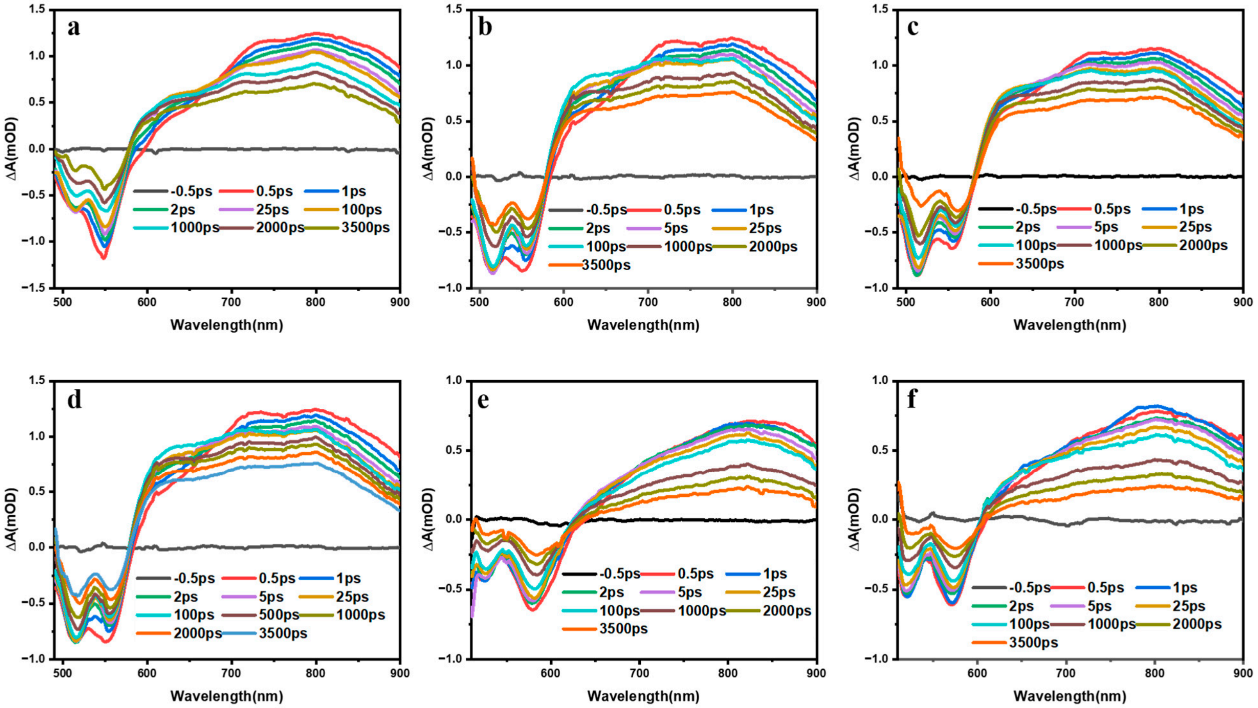


**Figure S2. The transient absorption spectra of the PDI trimmer at all time delays with different water percentages: a:0%; b:10%; c:20%; d:30%; e:40%; f:50%.**

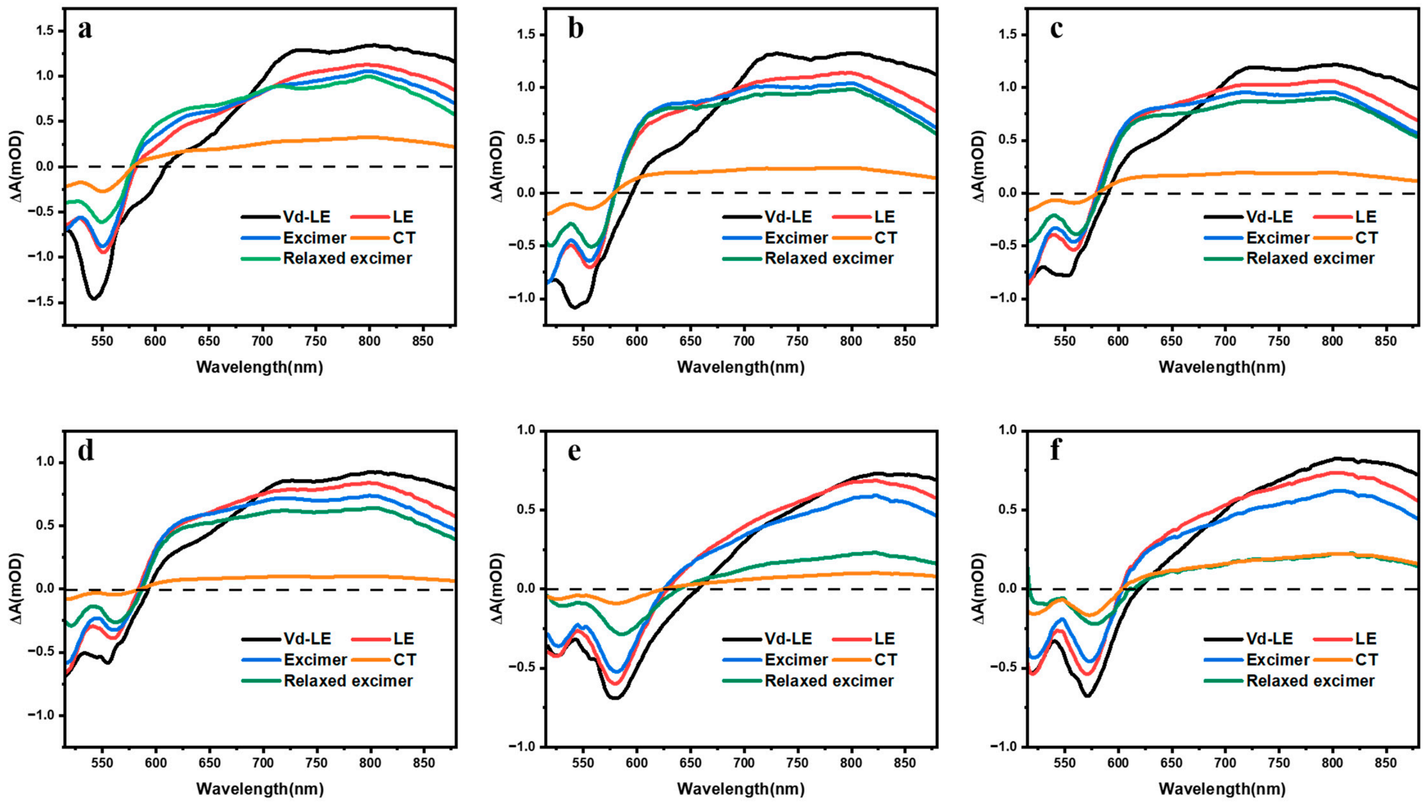


**Figure S3. SADS data of different species with different water percentages: a:0%; b:10%; c:20%; d:30%; e:40%; f:50%.**

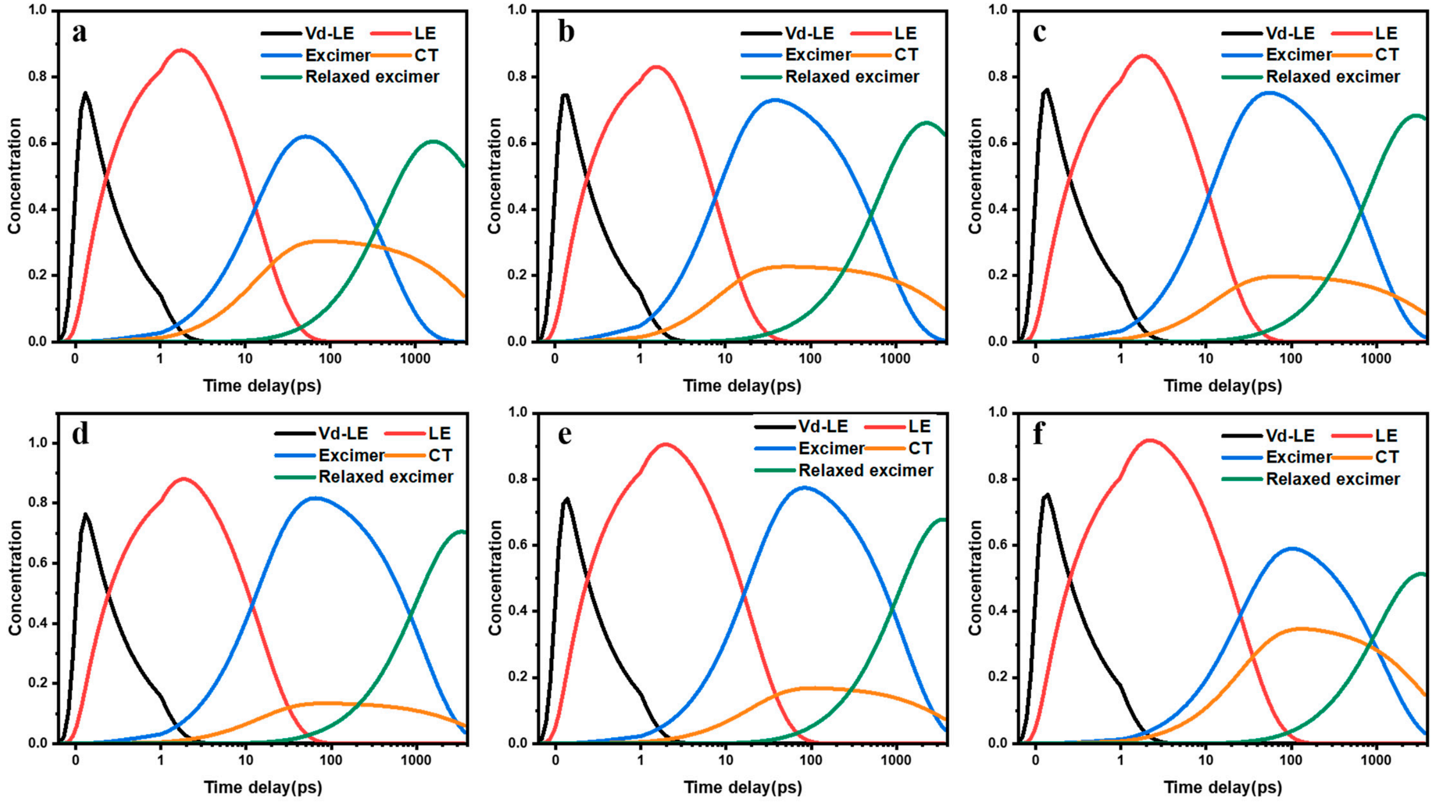


**Figure S4. The concentration variations of corresponding processes when different water percentages: a:0%; b:10%; c:20%; d:30%; e:40%; f:50%.**
